# Supplementary material for: Content-rich biological network constructed by mining PubMed abstracts
Source: BMC Bioinformatics. 2004 Oct 8;5:147. doi: 10.1186/1471-2105-5-147 (PMC528731; doi:10.1186/1471-2105-5-147)
Supplement: Additional File 2 — The original results of the above study (non-essential files are deleted to keep the file size under the limit set by BMC bioinformatics). [file 1471-2105-5-147-S2.bz2 › chilibotAdditionalFile2/dip20/401ID7539796E9997/html/CNTF_CNTFR.html]

 


 **CNTF** and **CNTFR** 
  
Found 97 abstracts in PubMed, retrieved 20.  
 

 What does Google say? 
 PDF only 
| .edu only 

---

**Interactive relationship** (e.g. stimulation, inhibition, etc)

**Inhibitory relationship**- The functional receptor complex of  **CNTF**  is composed of the  **CNTF**  receptor alpha  **CNTFR** , gp130 and the leukemia inhibitory factor receptor LIFR.  Ref: 11943154 FEBS Lett, 2002
- Human  **CNTF**  elicits cellular responses by induction of a receptor complex consisting of the  **CNTF**  alpha receptor  **CNTFR** , which is not involved in signal transduction, and the beta receptors gp130 and leukemia inhibitory factor receptor LIFR.  Ref: 12643274 J Biol Chem, 2003
- Human  **CNTF**  elicits cellular responses by induction of a receptor complex consisting of the  **CNTF**  a receptor  **CNTFR** , which is not involved in signal transduction, and the b receptors gp130 and leukaemia inhibitory factor receptor LIFR.  Ref: 12517918 J Biol Chem< MedlineTA>J Biol Chem, 2003
- The functional receptor complex of ciliary neurotrophic factor  [ **CNTF** ]   **CNTF** , a member of the gp130 family of cytokines, is composed of  **CNTF** , the  **CNTF**  receptor alpha  **CNTFR** , gp130 and the leukemia inhibitory factor receptor LIFR.  Ref: 12707266 J Biol Chem< MedlineTA>J Biol Chem, 2003
- For signal transduction,  **CNTF**  requires a receptor complex, composed of the IL 6 signal transducing molecule gp130, leukemia inhibitory factor receptor LIFR beta, and  **CNTFR**  alpha  [ **CNTFR** ] .  Ref: 11464862 Med Pediatr Oncol, 2001
**Neutral relationship**- Engineering a  **CNTFR**  specific human  **CNTF**  variant may therefore be a prerequisite to improve the safety profile of  **CNTF** .  Ref: 12517918 J Biol Chem< MedlineTA>J Biol Chem, 2001
- Engineering a  **CNTFR**  specific human  **CNTF**  variant may therefore be a prerequisite to improving the safety profile of  **CNTF** .  Ref: 12643274 J Biol Chem, 2003
- These data suggest that both the gp130 CBD and the LIFR CBD1 interact with  **CNTFR**  in a similar way and provide insights into the nature of the  **CNTF**  receptor complex.  Ref: 12707266 J Biol Chem< MedlineTA>J Biol Chem, 2003

**Non-interactive relationship** (e.g. studied together, co-existance, homology, etc.)

- The mRNA levels of neuropoietic cytokines, ciliary neurotrophic factor  [ **CNTF** ]   **CNTF** , leukemia inhibitory factor LIF, and interleukin 6 IL 6, and their receptor components  **CNTFR**  alpha  [ **CNTFR** ] , LIFR beta, IL 6R alpha, and gp130 were examined in seventy six patients with various peripheral neuropathies to determine the extent of expression of these cytokines and receptors, and their relationship to nerve fiber pathology and cell infiltration in the diseased nerves.  Ref: 11358282 Neurochem Res, 2001
- We showed that ciliary neurotrophic factor  [ **CNTF** ]  receptor  **CNTFR**  gene expression was higher in muscle biopsy tissue from severely affected MG patients regardless of anti nAChR antibody titer.  Ref: 11694333 J Neuroimmunol, 2001
- This study reinforces the functional similarity between  **CNTF**  and the CLC CLF composite cytokine defining the second ligand for  **CNTFR** .  Ref: 11294841 J Biol Chem, 2001
- In contrast to that reported before for  **CNTF** , soluble  **CNTFR**  failed to promote the action CLC CLF, and an absolute requirement of the membrane form of  **CNTFR**  was required to generate a functional response to the composite cytokine.  Ref: 11294841 J Biol Chem, 2001
- The C174T polymorphism in the  **CNTF**  receptor  **CNTFR**  gene is associated with fat free mass in men and women.  Ref: 12807897 J Appl Physiol< MedlineTA>J Appl Physiol, 2001
- Expression of mRNAs for ciliary neurotrophic factor  [ **CNTF** ]   **CNTF** , leukemia inhibitory factor LIF, interleukin 6 IL 6, and their receptors  **CNTFR**  alpha  [ **CNTFR** ] , LIFR beta, IL 6R alpha, and gp130 in human peripheral neuropathies.  Ref: 11358282 Neurochem Res, 2001
- Continuous treatment with Dex 10 8 mol L inhibited the endogenous production of IL 6, LIF, OSM, IL 11R, and OSMR, but had no detectable effect on the expression of IL 11, CT 1,  **CNTF** ,  **CNTFR** , LIFR, or gp130.  Ref: 12110437 Bone, 2002
- We previously reported that the membrane distal cytokine binding domain CBD1 of LIFR could interact in vitro with soluble  **CNTFR**  sCNTFR in the absence of  **CNTF** .  Ref: 12707266 J Biol Chem< MedlineTA>J Biol Chem, 2002
- We performed gene screening of the ciliary neurotrophic factor  [ **CNTF** ]  receptor  **CNTFR**  gene and genotyped three newly identified polymorphisms C 1703T in the 5 promoter region, T1069A in intron 5, and C174T in exon 9.  Ref: 12807897 J Appl Physiol< MedlineTA>J Appl Physiol, 2002
- Given the restricted expression domain of the  **CNTF**  receptor alpha  **CNTFR**  in the developing forebrain germinal zone and adult forebrain periventricular area, we have examined the putative role of  **CNTFR**  LIFR gp130 mediated signaling in regulating forebrain neural stem cell fate in vivo and in vitro.  Ref: 11567054 J Neurosci, 2001
- In addition, the expression of ciliary neurotrophic factor  [ **CNTF** ]  receptor  **CNTFR**  and leukemia inhibitory factor receptor LIFR mRNAs, but not of interleukin 6 receptor mRNA, was up regulated after systemic lipopolysaccharide treatment.  Ref: 12127095 Neuroscience, 2002
- Striking phenotypic differences between  **CNTF**  and  **CNTFR**  deficient mice suggest that  **CNTFR**  serves as a receptor for a second developmentally important ligand.  Ref: 11294841 J Biol Chem, 2001
- In this study, we provide the first demonstration that the membrane distal CBD CBD1 of LIFR associates in vitro with soluble  **CNTFR**  in the absence of  **CNTF** .  Ref: 11943154 FEBS Lett, 2002
